# Differential correlation for sequencing data

**DOI:** 10.1186/s13104-016-2331-9

**Published:** 2017-01-19

**Authors:** Charlotte Siska, Katerina Kechris

**Affiliations:** 1 Computational Bioscience Program, Department of Pharmacology, School of Medicine, University of Colorado Anschutz Medical Campus, Aurora, CO USA; 2 Department of Biostatistics and Informatics, Colorado School of Public Health, University of Colorado Anschutz Medical Campus, Aurora, CO USA

## Abstract

**Background:**

Several methods have been developed to identify differential correlation (DC) between pairs of molecular features from –omics studies. Most DC methods have only been tested with microarrays and other platforms producing continuous and Gaussian-like data. Sequencing data is in the form of counts, often modeled with a negative binomial distribution making it difficult to apply standard correlation metrics. We have developed an R package for identifying DC called Discordant which uses mixture models for correlations between features and the Expectation Maximization (EM) algorithm for fitting parameters of the mixture model. Several correlation metrics for sequencing data are provided and tested using simulations. Other extensions in the Discordant package include additional modeling for different types of differential correlation, and faster implementation, using a subsampling routine to reduce run-time and address the assumption of independence between molecular feature pairs.

**Results:**

With simulations and breast cancer miRNA-Seq and RNA-Seq data, we find that Spearman’s correlation has the best performance among the tested correlation methods for identifying differential correlation. Application of Spearman’s correlation in the Discordant method demonstrated the most power in ROC curves and sensitivity/specificity plots, and improved ability to identify experimentally validated breast cancer miRNA. We also considered including additional types of differential correlation, which showed a slight reduction in power due to the additional parameters that need to be estimated, but more versatility in applications. Finally, subsampling within the EM algorithm considerably decreased run-time with negligible effect on performance.

**Conclusions:**

A new method and R package called Discordant is presented for identifying differential correlation with sequencing data. Based on comparisons with different correlation metrics, this study suggests Spearman’s correlation is appropriate for sequencing data, but other correlation metrics are available to the user depending on the application and data type. The Discordant method can also be extended to investigate additional DC types and subsampling with the EM algorithm is now available for reduced run-time. These extensions to the R package make Discordant more robust and versatile for multiple –omics studies.

**Electronic supplementary material:**

The online version of this article (doi:10.1186/s13104-016-2331-9) contains supplementary material, which is available to authorized users.

## Background

Differential correlation or coexpression (DC) occurs when two features show dissimilar associations between biological groups. DC has been gaining ground as another approach to analyze –omics data, especially when individual features may not show differential expression or abundance, but may be differentially associated among groups, indicating a potential biological interaction [1]. DC has been previously examined in both low and high-throughput studies. For example, it was determined using chromatin immunoprecipitation that the mutated form of p53 reduces the binding of wild-type p53 to the p53 response element p21. This mutation results in DC of p53 with its target genes MDM2 and PIG3, when comparing wild-type p53 with mutant p53 [2]. In another study, interleukins and tumor necrosis factor were DC between patients with untreated and treated paracoccidioidomycosis using ELISA and lymphoproliferation assays [3]. In addition, a recent transcriptomics study examined expression differences between lean and obese siblings and found that NEGR1 is a central hub in obesity-related DC networks [4].

Discordant, a DC method based on mixture models, has exclusively been tested on microarray and other platforms that produce continuous and Gaussian-like data [5]. Several other methods have also only been validated using microarray data, but not on sequencing data generated from next-generation sequencing technologies [6, 7, 8]. This is most likely because sequencing technologies are relatively recent compared to microarray platforms and they result in count data so that standard correlation statistics may not be applicable. The challenge is best represented in comparing the methods for differential expression in microarrays vs. RNA-Seq. In microarrays, a student *t* test for each gene, or Empirical Bayes variation, will suffice [9] while RNA-Seq analysis often relies on negative binomial modeling [10–12].

Efforts to find a single best metric to measure correlation for –omics data is inconclusive because performance depends on the distribution of the data, sample size and the observation of interest [13]. For example, Pearson’s correlation can be used when the distribution is approximately Gaussian and the user has interest in linear relationships, while Hoeffing’s D measure may be better suited for non-monotonic associations [13]. Two studies compared correlation statistics for –omics data but were validated using microarrays [13, 14]. In our work, we examine a variety of correlation statistics appropriate for sequencing data for the purpose of testing DC including Pearson’s, Spearman’s, biweight midcorrelation (BWMC) and a sparse compositional correlation method SparCC [15].

There are two R packages available for DC, DiffCorr and EBcoexpress [7, 16]. These packages also provide functions to create graphs or networks of the data in addition to gene module analysis. To complement these existing packages, our work also introduces a new R package called Discordant. The Discordant package has been developed not only for sequencing data but with additional extensions to make the DC analysis more flexible and usable.

One of the extensions provided in Discordant is to increase the observable DC classes. Currently, the only types of DC observed are cross (associations is in opposite directions between groups) or disrupted (association is present in one group but not the other). Previous experimental studies have observed cases of other types of DC when there is an increase in association in one group versus the other. For example, antigen coexpression increased in women 3 days after vaginal delivery [17] and eotaxin and interleukin-5 coexpression was increased in blister fluid of patients with bullous pemphigoid compared to healthy patients [18]. These subtle increases (or decreases in association) are more difficult to study in current DC packages and we have made that option available in Discordant. The other extension provided decreases run-time when millions of feature pairs may be evaluated. The number of pairs is especially relevant to data generated from sequencing technologies since the feature size (genes, miRNA, etc.) can be larger than data generated from microarray platforms [19].

In summary, we have included several extensions to the Discordant method in a new R package that broadens applicability: (1) different correlation metrics for sequencing data, (2) more differential correlation classes and (3) a subsampling approach. Simulations and The Cancer Genome Atlas (TCGA) breast cancer sequencing data are used to compare performance of the correlation methods and extensions.

## Implementation

The Discordant model is adapted from Lai et al algorithm to determine concordance between microarrays [20, 21]. The model has been previously published [5].

**Fig. 1:**
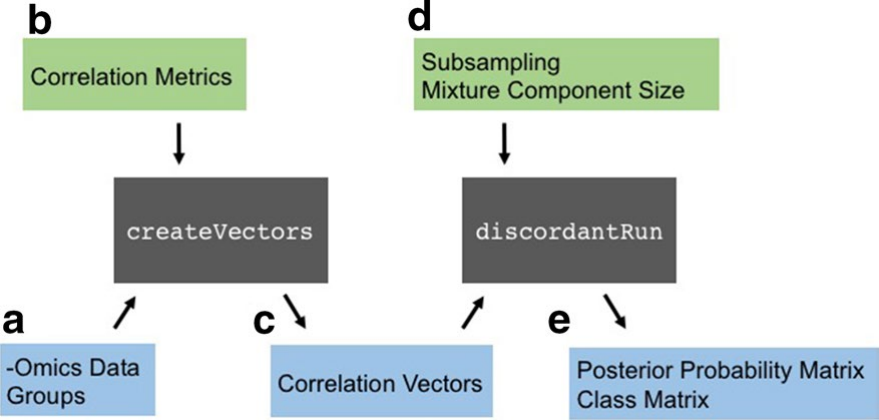
Flow of Discordant R Package. *Gray boxes* are functions, *blue boxes* outputs and *green boxes* optional parameters. **a** Input argument for createVectors(). **b** Optional argument for createVectors(). **c** Output argument for createVectors(), input for discordantRun(). **d** Optional arguments for discordantRun(). **e** Outputs for discordantRun()

The R package for the Discordant method was designed for user flexibility. It is separated into two main functions: createVectors and discordantRun. The flow of these functions are illustrated in Fig. 1 and described further below.

### –Omics data as mandatory input for each function

Each function requires either one or two –omics dataset(s) as argument(s). If only one –omics dataset is used as an argument, the functions undergo a within –omics analysis, where pairs of the same feature are evaluated (e.g. gene–gene, metabolite–metabolite). If two –omics datasets are used, the functions undergo a paired –omics analysis, where pairs of different features are evaluated (e.g., gene-miRNA, gene-metabolite).

#### createVectors

The purpose of createVectors is to determine correlation coefficients. The inputs are the –omics dataset(s) and a group vector that indicates samples for groups 1 and 2 (Fig. 1a). An optional parameter called cor.method is available where the user can change the type of correlation (Fig. 1b). The options are Pearson, Spearman, BWMC and SparCC (described in detail below). The default value for cor.method is Pearson. The output are two correlation vectors corresponding to the biological groups (Fig. 1c).

#### Correlation metrics

Four correlation metrics are applied to sequencing data: Pearson, Spearman, Biweight midcorrelation (BWMC) and SparCC. The commonly used Pearson correlation assumes that both data vectors are normally distributed, and is optimal for identifying linear relationships. Spearman correlation is rank-based and a non-parametric alternative that can handle non-normal data and capture monotonic and linear relationships. BWMC is much like Pearson’s correlation, except it is median-based rather than mean-based. It is considered more robust than Pearson’s correlation method since it minimizes the effect of outliers on the final correlation [22]. SparCC (Sparse Compositional Correlation) correlations are approximated based on the dispersion of the data and the assumption that most feature pairs will have no correlation [15].

#### discordantRun

The arguments necessary for discordantRun are the correlation vectors and –omics dataset(s) (Fig. 1c). The correlation vectors are input for the Discordant algorithm, and the –omics dataset(s) are used to communicate whether a single –omics or double –omics analysis is requested. The default value for transform is TRUE, which transforms correlation vectors into z scores using Fisher’s transformation. This option was inserted into discordantRun instead of createVectors so users could generate correlation vectors independent of the R package.

Optional arguments are available in order to use the 5-component mixture model or subsampling (Fig. 1d). The outputs are a posterior probability matrix and class matrix (Fig. 1e). For single –omics analyses, results in the output matrices are below the diagonal of the matrix and NAs are above the diagonal in order to avoid duplicating information. The posterior probability output is the differential correlation posterior probabilities (the sum of the off-diagonal in the class matrix in Additional file 1: Figure S1d).

There are several outputs for discordantRun: discordPPMatrix, discordPPVector, classMatrix, classVector, probMatrix, and loglik. The outputs discordPPMatrix and discordPPVector contain the posterior probability of differential correlation. The rows of discordPPMatrix are the features in –omics x and the columns are the features in –omics y (or x if within –omics analysis is being used). The class that had the highest probability for each feature pair are contained in classMatrix and classVector. The formatting of classMatrix is similar to discordPPMatrix. All complete information is in probMatrix, where each row represents a feature pair and nine columns represent the class within the class matrix. The log likelihood of the data fitting the model is in the argument loglik.

#### Comparison to DiffCorr

The analysis of sequencing data by Discordant was compared to DiffCorr, an R package that uses Fisher’s method to determine differential correlation [7]. Correlation vectors were determined using createVectors() and then DiffCorr’s function compcorr() was used to calculate p values. Simulations and biological validations were used to assess performance.

#### 5-Component normal mixture model

In the simplest model [5], a three component mixture model is used to define whether correlations are not present (0), are positive (+) or are negative (−). We offer an option which increases the number of components to 5, adding correlations that are very positive (++) or very negative (−). This increases the parameter size from 21 to 35 and the number of classes from 9 to 25 (Additional file 1: Figure S2). To run the Discordant algorithm with 5 components instead of 3, set parameter components to 5. The default value for components is 3.

#### Subsampling

Like other methods [6], the Discordant model makes a false assumption that molecular feature pairs are independent of each other, but features are in multiple pairs which violates the independence assumption. A subsampling option is included to address the assumption and also cut down run-time. Subsampling will run if the argument subsampling is set to TRUE. By default, the EM algorithm updates parameter estimates across all molecular feature pairs until the EM algorithm converges [23]. With the subsampling option, a subsample of correlation coefficients independent of each other are sampled to run the EM algorithm. This is repeated for a number of iterations (default is 100), and the parameters of each mixture component from each iteration are summarized by their mean (Additional file 1: Figure S3 a–e). Once the summarized parameters of the mixture components are determined, the posterior probabilities of all molecular features are determined (Additional file 1: Figure S3 f).

### TCGA breast cancer data

From the Cancer Genome Atlas (TCGA, http://cancergenome.nih.gov), we accessed miRNA-Seq and RNA-Seq breast cancer data with matched subjects. Of these, there are 15 samples with normal (or control) tissue and 42 samples with tumor tissue. This dataset was selected because it had the largest sample size of control and tumor groups in TCGA that had matched samples with miRNA-Seq and RNA-Seq data. Both datasets were pre-processed and normalized using HTSeq filtering and TMM normalization [24, 25]. For Peason’s correlation and BWMC [9] the data was also transformed using voom from the limma R package to create continuous values [26], otherwise the methods (Spearman and SparCC) were applied to normalized count data. The number of features remaining were 212 miRNA and 19414 mRNA.

Features were further filtered by the presence of outliers using the median absolute deviation (MAD) outlier method [27]. Even after pre-processing and normalization, the distribution of sequencing data still is asymmetrical, where there is large density around zero and long tails to the right. To determine outliers, the values for each feature are split by being greater or less than the median. The two sets of features are tested for outliers by the difference they have with their respective MAD [28]. The maximum distance of all features from the upper or lower MAD is used to determine if the feature has an outlier. The standard threshold is two or three times the MAD outside the median [27], but since we found that the variation in the sequencing data was more extreme we used larger thresholds. For the transformed sequencing data, a threshold of 7 is used to retain features but still filter out those that were most problematic. Non-transformed data has even larger dispersions, so a threshold of 20 was used. The number of features after filtering for outliers is 16,656 for RNA-Seq data and 200 for miRNA-Seq data for the voom-transformed data and 17,972 for RNA-Seq and 200 for miRNA-Seq for non-transformed data . The code for this outlier method is included as a function called madOutlier in the R package with an option to change the threshold.

Experimentally validated breast cancer miRNA not involved in other well-researched cancers (prostate cancer, melanoma, glioblastoma multiforme) were used as a biological validation [29, 30]. A total of 8 unique breast cancer miRNAs were found to be in the TCGA breast cancer data. Since the result are in the form of molecular feature pairs, breast cancer miRNAs occur in more than one pair. Therefore, for comparison purposes we report the first occurrence of the miRNA in the pairs ranked by posterior probability.

## Results

### Correlation methods comparison

#### Simulations

**Fig. 2:**
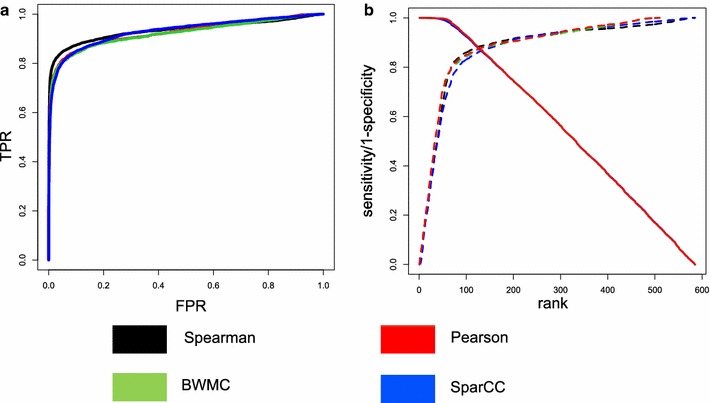
Comparisons of correlation methods: **a** ROC, **b** Sensitivity/1-Specificity vs. rank

The simulation designs are explained in Additional material and Additional file 1: Figure S4. Four correlation metrics (Spearman, Pearson, SparCC, BWMC) were applied to simulated data to assess performance in identifying DC molecular feature pairs. Two methods were applied to count data (Spearman, SparCC) and two methods were applied to transformed data (Pearson, BWMC). Spearman’s correlation was used in DiffCorr. Figure 2a, b shows the sensitivity and specificity of the methods.

Pearson and BWMC perform similarly, most likely because their models are similar except Pearson is mean-based and BWMC is median-based (Fig. 2a, b). SparCC has higher performance than all other methods except for Spearman. Out of all correlation metrics, Spearman had greater area under the curve in both the ROC curve and the sensitivity curve. Since Spearman’s correlation was the metric that had the best performance, it is used in the simulations and biological validation for the evaluations of the extensions (5-component and subsampling).

#### TCGA breast cancer data

To identify miRNA–mRNA pairs that may change interactions due to tumor status, we evaluated miRNA and mRNA sequencing data from the TCGA database for breast cancer. Discordant was run with four different correlation metrics (Spearman, Pearson, BWMC and SparCC). In Table 1, the ranks and probability of the most significant pairing of a breast cancer miRNA with a gene is summarized from Additional file 1: Table S1. For almost all our benchmark breast cancer miRNA, Spearman correlation finds them to be more highly significant than any other method. The results are similar to those in simulations (Fig. 2a, b) except for SparCC. In the simulations, SparCC performed second to Spearman but in the biological validation it performs worse except for Pearson.

**Table 1:** Summarized TCGA breast cancer biological validation

Treatment	Mean rank	Median rank	Mean q value/FDR
Correlation method comparison			
Spearman	89	56	0.0150
SparCC	543	387	0.0240
Pearson	531	512	0.0600
BWMC	294	275	0.0530
DiffCorr	502	395	0.2100
3 components vs. 5 components			
3	89	56	0.0150
5	683	392	0.0007
Standard EM vs. subsampling EM			
Standard EM	89	56	0.0150
Subsampling EM	92	44	0.0048

Ranks are summarized by mean and median, and q values/FDR are summarized by mean. Values here are based on values in Additional file 1: Table S1

### Comparison to DiffCorr

To compare both DiffCorr and Discordant, Spearman’s correlation metric was used because it demonstrated better performance in the correlation metric comparison. Using Spearman’s correlation, the two methods were similar in the simulations, with a slight advantage for Discordant, which displayed more area under the curve than DiffCorr (Additional file 1: Figure S4). Pearson’s correlation applied to DiffCorr was also observed, and showed worse performance than Spearman’s correlation applied to DiffCorr (data not shown). However, the difference with DiffCorr is more evident in the TCGA Breast Cancer data, where breast cancer miRNAs in the DiffCorr analysis showed much higher rank and FDR compared to Discordant (Table 1; Additional file 1: Table S1).

### 5-Component normal mixture model

#### Simulations

The 3-component normal mixture model was compared to the 5-component mixture model using Spearman’s correlation, which performed best in the simulations described above. Since the 3-component mixture model does not have components for −− and + +, any class that has a −− or ++ is assumed to be − and + respectively, and the status as a true positive or true negative is assigned appropriately. The extra classes introduced by the 5-component mixture model are elevated DC, which is when the association in one group is stronger than in the other group. The elevated DC classes are true positives in the 5-component mixture model, but in the 3-component mixture model, these classes are designated as true negatives.

**Fig. 3:**
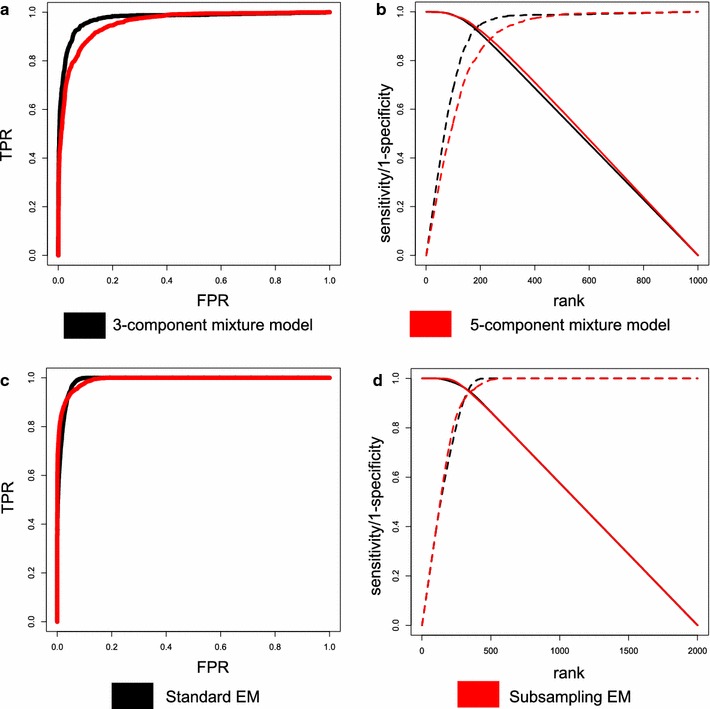
Analysis of extensions. 3-Component mixture model vs. 5-component mixture model: **a** ROC, **b** Sensitivity/1-specificity vs. rank. Standard EM vs. Subsampling EM: **c** ROC, **d** Sensitivity/1-specificity vs. rank

The 3 component normal mixture model has greater power than 5-component mixture model (Fig. 3a, b). The plots of the posterior probabilities of true positives and true negatives for the simulations show (Additional file 1: Figure S6a, b) that the 5-component mixture models are better at identifying true positives but clearly struggle with true negatives. In Additional file 1: Figures S7–S10 the distribution of posterior probabilities for DC are plotted for each class, and it is evident that 5 component mixture model struggles to make distinctions between the − and −− components and the + and ++ components. In the elevated DC case (−/−− and +/++) we expect high posterior probabilities in the 5-component mixture model but low posterior probabilities in the 3-component mixture model. The posterior probabilities for 5-component are higher, but they have wide range whereas the posterior probabilities for the 3-component mixture model are close to 0 with tight distribution (Additional file 1: Figure S9). A similar situation is in the cases where there is no DC (Additional file 1: Figure S10), which we expect low posterior probabilities for both 3-component and 5-component mixture models. In the classes ++/++ and −−/−− the distributions for the 5-component mixture model are variable whereas the distributions for the 3-component mixture model are close to 0 with low variation.

The average run-time for the simulations were also determined, and as expected the 5-component mixture model takes longer to finish (by a factor of 3) since there are more parameters (Additional file 1: Table S2). Although our simulations show reduced performance, this option is included for users interested in these types of associations.

#### TCGA breast cancer data

Table 1 shows the summarized ranks and posterior probability of the most significant breast cancer miRNA gene pair when Discordant is run with the 3- or 5-component mixture models (complete information in Additional file 1: Table S1). In terms of rank, the 3-component mixture model does better but the 5-component mixture models have higher posterior probabilities. The final posterior probability is a summation of all DC posterior probabilities (Additional file 1: Figure S1f). For the 3-component mixture model there are 6 out of 9 of the posterior probabilities for the DC classes, whereas for 5 component mixture models there are 20 out of 25. The posterior probabilities for 5-component mixture models may be larger because a greater proportion of the total class posterior probabilities are used to summarize the final DC posterior probability.

### Subsampling

#### Simulations

The standard EM algorithm and subsampling version of the EM Algorithm were compared to assess affects performance. The ROC curves and the sensitivity/specificity are similar for the two versions (Fig. 3c, d). The distribution of posterior probabilities of True Positives (TP) and True Negatives (TP) for the subsampling version were lower for the TN and higher for TP, indicating greater power (Additional file 1: Figure S6c, d). This is also evident when looking at each class posterior probabilities separately (Additional file 1: Figures S11–S13). In addition, an advantage of the subsampling version is that the run-time decreases by a factor of two (Additional file 1: Table S2).

#### TCGA breast cancer data

We evaluated the results from the subsampling version on the TCGA Breast Cancer data. The summary in Table 1 and details in Additional file 1: Table S1 show that the subsampling with EM has similar ranks for breast cancer miRNAs, but higher posterior probabilities than the standard EM implementation.

## Discussion

Biological data generated from high-throughput experiments do not always have a normal distribution. It is critical to evaluate models to ensure they are able to make accurate predictions with non-normal data. The most common example of non-normal data is that from sequencing, which is becoming the platform of choice [31]. In this study, we have demonstrated that the Discordant method is now applicable to sequencing data and other platforms that produce discrete or count data. Correlation metrics were compared in simulations and TCGA breast cancer data. BWMC performs similarly to Pearson but demonstrated more power in the biological validations. This may be because BWMC is more robust to the presence of outliers and non-symmetric distributions.

SparCC demonstrated better performance in the simulations compared to the biological validation. In the simulations, the correlated mRNAs to miRNAs were generated based on the mean of the miRNA and the dispersion of the mRNA. The distributions were more similar in the correlated pairs in the simulations than the feature pairs in the biological validation since their distributions shared similar parameters. SparCC predicts the actual values based on the dispersion of the observed values, therefore using two different types of –omics with their own unique variation may not be suitable.

Spearman’s correlation metric demonstrated the best performance compared to all other metrics in both the simulations and the biological validation. Spearman’s correlation is a non-parametric rank-based metric that makes it well suited for non-normal distributions. Using non-parametric methods when integrating datasets with different levels of variability is favorable, and may explain why Spearman has greater power in both simulations and biological validation.

Although we found improved performance with Spearman’s correlation in our simulations and sequencing data, the preferred correlation metric depends on the type of data and study. For example, if the user wants a more conservative method SparCC should be used, but for normal continuous data Pearson’s correlation is the natural choice. For these reasons, the correlation metric is a user-defined option in the Discordant R package.

DiffCorr and Discordant were compared to assess the application of sequencing data to another published differential correlation R package. Spearman’s correlation metric was used since it demonstrated better performance compared to other metrics when applied to Discordant. It was not surprising that Discordant demonstrated better performance since we have shown in previous studies that Discordant’s model outperforms the Fisher's method DiffCorr implements [5, 7, 32]. If DiffCorr is a more attractive option based on its computational simplicity compared to Discordant, sequencing data can be applied with the Spearman’s correlation metric.

Not only does the new Discordant R package provide more flexibility to the form of the data, it also provides additional modeling and implementation options compared to existing software DiffCorr and EBcoexpress [7, 16]. Expanding the normal mixture model from 3 components to 5 components gives the user the opportunity to explore other types of DC, which have been documented in several low-throughput experiments before, and may be of interest to other researchers [17, 18]. The addition of extra classes does reduce power for Discordant, and we advise users to consider the 5-component mixture model if extreme DC is relevant to their study and a 5-component mixture model is justifiable with model selection criteria such as Bayesian Information Criteria (BIC).

Furthermore, users are now able to use the subsampling extension to the EM algorithm which not only makes the method more computationally tractable, but also solves the problem of dependencies between pairs. There were some inconsistencies, such as the higher posterior probabilities for subsampling compared to the posterior probabilities with no subsampling even though the ranks between subsampling and no subsampling were similar. The posterior probabilities may be larger for two reasons: the parameters for each mixture component are better estimated since the independence assumption is no longer violated and the parameters are averaged over 100 iterations, making standard errors small. Users apply subsampling they should also be aware of selection bias, since only correlation coefficients that are independent of each other are used. Another limitation of subsampling is that the number of independent pairs is limited by the dimensions of the data. Finally, the theoretical properties of subsampling for the EM algorithm are not developed to our knowledge. Despite these considerations, our results illustrate the computational advantage of this extension without compromising performance.

Both DiffCorr and EBcoexpress have visualizations in their R packages, but visualizations were not included in the Discordant R package because plots can be made with added-in functions or additional libraries such as plot() and ggplot2 [33] and graphs can be made with R package igraph or GUI Cytoscape [34, 35]. DiffCorr has a module building option, which will be added to Discordant in the future. Module building is a complex future direction with multiple alternatives that needs to be thoroughly investigated to assess which application works best [36–42].

## Conclusions

Our expanded R package Discordant provides novel flexibility compared to previous versions and other software, with regards to data type, modeling options and implementation. To our knowledge the Discordant model may also be the first DC tool to be validated using negative binomial simulations and sequencing data. Sequencing data is arguably becoming the more common platform for transcriptomic and other data, therefore developing and testing new software to analyze sequencing data is essential.

## Additional file

**Additional file 1.** Additional figures and tables.

## Abbreviations

DC: differential correlation or coexpression
TCGA: The Cancer Genome Atlas
MAD: median absolute deviation
