# Supplementary material for: Differential correlation for sequencing data
Source: BMC Res Notes. 2017 Jan 19;10:54. doi: 10.1186/s13104-016-2331-9 (PMC5244536; doi:10.1186/s13104-016-2331-9)
Supplement: Supplementary file 1 — Additional file 1. Additional figures and tables. [file 13104_2016_2331_MOESM1_ESM.docx]

**Simulations**

Simulating sequencing data proves a challenge because there are no available R functions that simulate bivariate negative binomial data for a fixed covariance structure. Therefore, we used information from the TCGA breast cancer data to first generate a data set without any structure between miRNA and mRNA. Supplementary Figure 2 shows the pipeline to create simulations. First, parameters are generated based on the TCGA breast cancer data (Supplementary Figure 2a, step1) and then they are used to simulate data (Supplementary Figure 2b, step 2).

For each feature in the TCGA miRNA and mRNA sequencing data, we estimate the two group (control vs. tumor) means and common dispersion using R function glm.nb from library MASS (Supplementary Figure 2a).

| Step 1 | glm.nb(x_i~groups) → $\hat{\beta}$_0i_,$\hat{\beta}$_1i_,$\hat{\theta}$_i_  glm.nb(x_j~groups) → $\hat{\beta}$_0j_,$\hat{\beta}$_1j_,$\hat{\theta}$_j_ |
| --- | --- |

Variables x_i and x_j contains the counts for the i^th^ and j^th^ features in the miRNA and mRNA data sets respectively. The parameters for feature i are means $\hat{\beta}$_0i_ and $\hat{\beta}$_1i_ and dispersion $\hat{\theta}$_i_ and the parameters for feature j are means $\hat{\beta}$_0j_ and $\hat{\beta}$_1j_ and dispersion $\hat{\theta}$_j_. The variable groups signify which samples are in group 1 and group 2.

Next, the R function rnegbin and parameters generated from step 1 are used to simulate data, for each feature y (Supplementary Figure 2b).

| Step 2 | $\hat{\beta}$_0i_, $\hat{\theta}$_i_ → rnegbin → y_i1_  $\hat{\beta}$_0j_, $\hat{\theta}$_j_ → rnegbin → y_j1_  $\hat{\beta}$_0i_+$\hat{\beta}$_1i_, $\hat{\theta}$_i_ → rnegbin → y_i2_  $\hat{\beta}$_0j_+$\hat{\beta}$_1j_, $\hat{\theta}$_j_ → rnegbin → y_j2_ |
| --- | --- |

The variables y_i1_ and y_i2_ are the simulated features for group 1 and group 2 for the i^th^ miRNA feature and variables y_j1_ and y_j2_ are the simulated features for group 1 and group 2 for the j^th^ mRNA feature. A different random subset of 200 features of the miRNA and mRNA were used for each simulation to make it faster (Supplementary Figure 1c). For correlation metrics that required data to be transformed, voom transformation was applied to the counts once the final subset in Supplementary Figure 1c was determined.

*Dependence Simulation*

In step 3 (Supplementary Figure 2d) dependencies between the miRNA and mRNA are created. Generalized linear models were used to create mRNA features (correlated_mRNA) whose values were correlated with miRNA features. Positive and negative associations were included to capture both indirect and direct effects of miRNA on mRNA. Although not reflecting the canonical relationships of miRNA and mRNAs, positive correlations have been observed in previous studies (Pasquinelli, 2012). This was performed using the glm.nb function from library MASS with parameters from Step 2 where the mean is defined by y_i_ (the miRNA counts) and the dispersion $\hat{\theta}$_j_ to create 200 correlated pairs (Supplementary Figure 2d).

| Step 3 | mean = y_i_, dispersion = $\hat{\theta}$_j_ → rnegbin **→** correlated_mRNA |
| --- | --- |

Since the data have been normalized, no constant was used to scale the miRNA value in the generalized linear model. We use miRNA as the independent variable in the linear model since miRNA can target 3’UTR of genes and affect mRNA expression, and not vice versa (Cannell et al., 2008). This creates a subset of data with miRNA→mRNA relationships (orange squiggly lines in Supplementary Figure 1e), and we also include a set of independent mRNA (orange checkered pattern in Supplementary Figure 2e) which would be in miRNA-mRNA pairs that are not DC (i.e. non-correlated pairs that are the negative cases).

The data are then converted into correlation coefficients for each group (Supplementary Figure 1b). The highest correlations are swapped in the Fisher-transformed z vectors to match correlations in each the 9 classes in the class matrix of the Discordant model (Supplementary Figure 1d). These will be the true positives and there are 16 in each class.

Supplementary Figure 1. Discordant Pipeline (figure from Siska et al Bioinformatics (2015) btv633). (a) Pearson’s correlation coefficients for all –omics A and B pairs (b) Fisher’s transformation (c) Mixture model based on z scores (d) Class matrix describing between group relationships. Dark grey are cross DC, medium grey disrupted DC and white no DC (e) EM Algorithm used to estimate posterior probability (pp) of each class for each pair (f) Final output of DC pp for each pair.


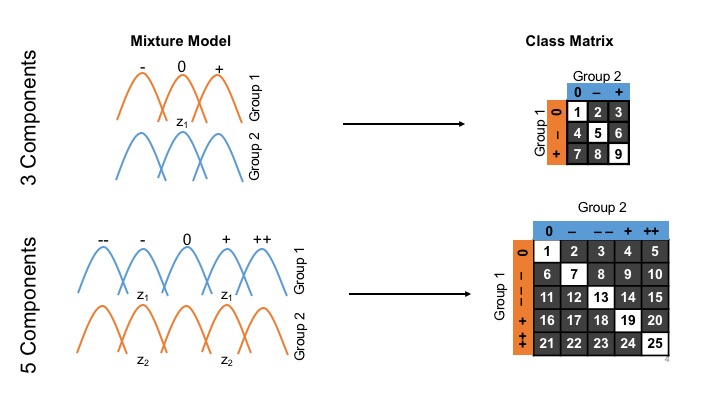


Supplementary Figure 2. Increasing from 3 to 5 components changes class matrix.


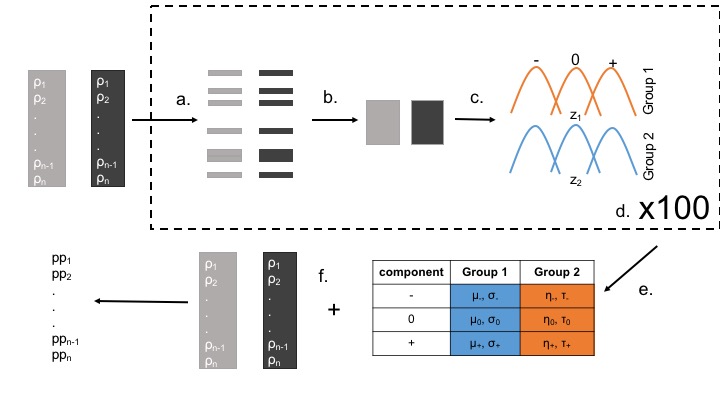


Supplementary Figure 3. Flow of subsampling. (a) Extract independent correlation coefficients. (b) Take independent correlation coefficients and create subset of correlation vectors. (c) Determine parameters of EM algorithm using subset of correlation coefficients. (d) Repeat steps a-c for 100 iterations. (e) Take average of parameters across runs. (f) Apply parameters to all features to obtain posterior probabilities (E-step of EM algorithm).


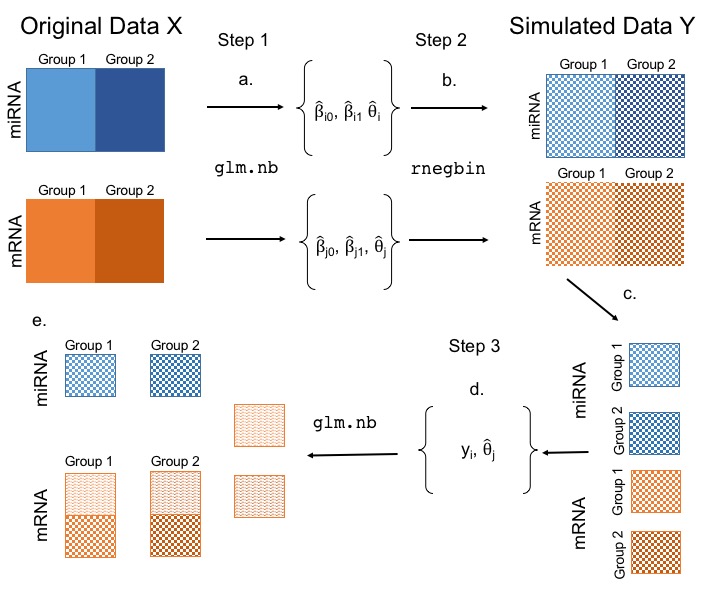


Supplementary Figure 4. Creating simulations from TCGA breast cancer data. (a) Determine θ and β of each feature using glm.nb and then use the same θ and β to create simulated data row by row with rnegbin (b) Choose randomly 200 pairs from miRNA and 200 pairs from RNA (c) Simulate mRNA features that are positively or negatively correlated to miRNA (d) Stack generated mRNA features on top of mRNA simulated subset.


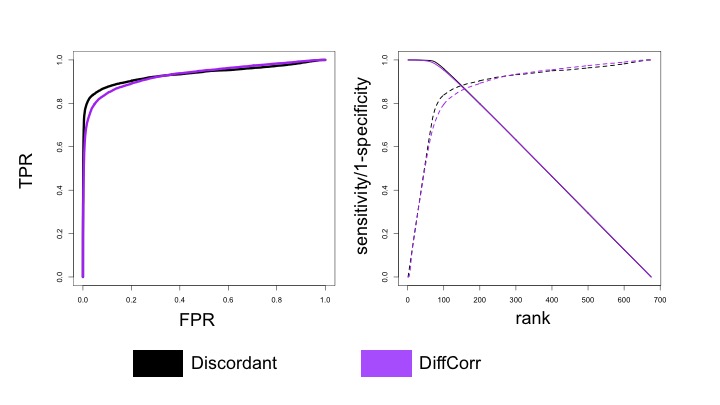


Supplementary Figure 5. Comparison of Discordant to DiffCorr R package using Spearman’s correlation metric. (a) ROC, (b) Sensitivty/1-Specificity vs. rank.


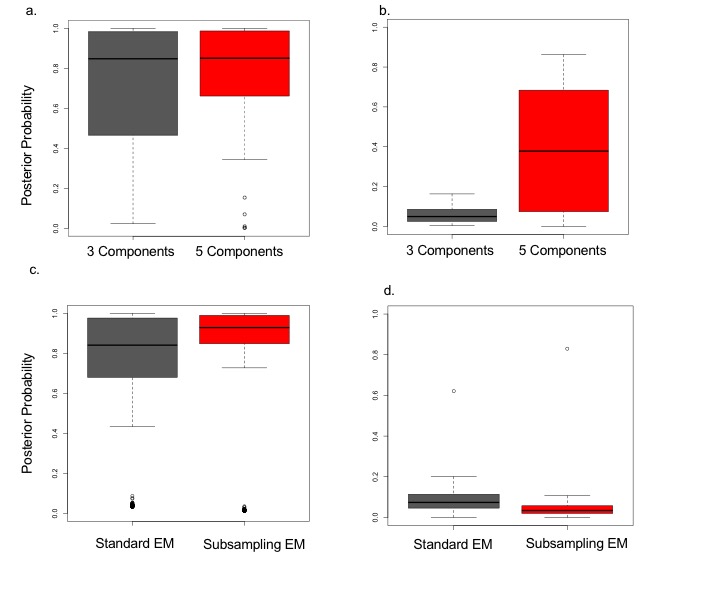


Supplementary Figure 6. Posterior probability distributions of true negatives and true positives for 5-components vs. 3-components and Standard vs. Subsampling EM. (a) True negatives of 5-components vs. 3-components. (b) True positives of 5-components vs. 3-components. (c) True positives of standard EM vs. subsampling EM. (d) True negatives of standard EM vs. subsampling EM.


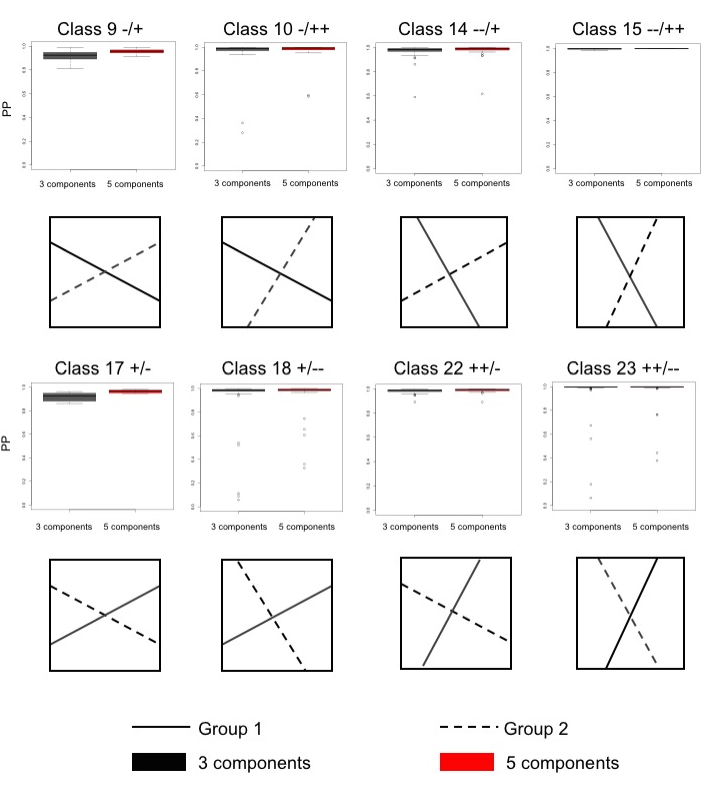


Supplementary Figure 7. Posterior probability distribution of cross DC in 3- vs. 5- components. Since these are cases of DC, the posterior probability of DC should be skewed to values close to 1.

**
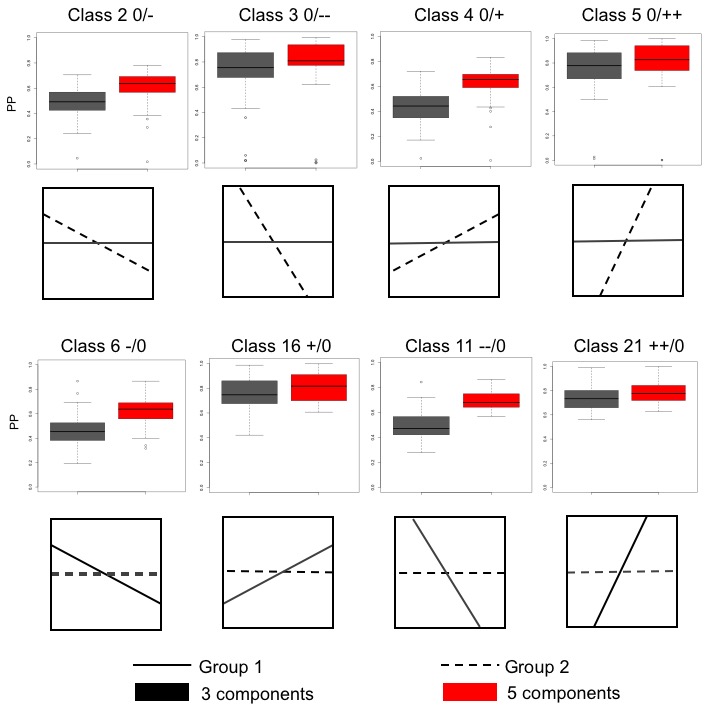
**

Supplementary Figure 8. Posterior probability distribution of disrupted DC in 3- vs. 5-components. Since these are cases of DC, the posterior probability of DC should be skewed to values close to 1.


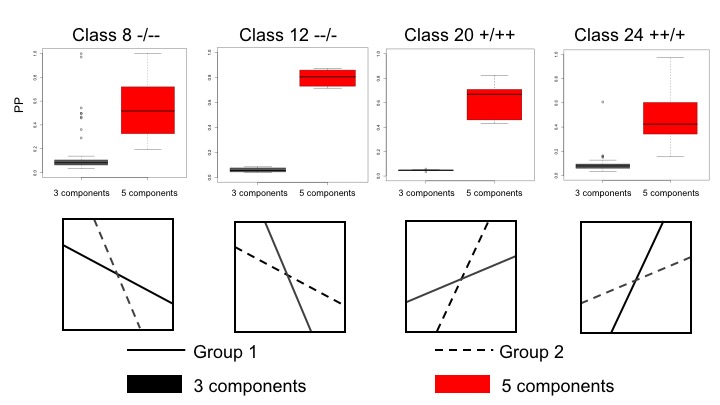


Supplementary Figure 9. Posterior probability distribution of elevated DC in 3- vs. 5- components. Since these cases are not modeled in the 3-component model, the posterior probability of DC should be skewed to values close to 0. In contrast, the 5-component model includes these cases and the posterior probability of DC should be skewed to values close to 1.


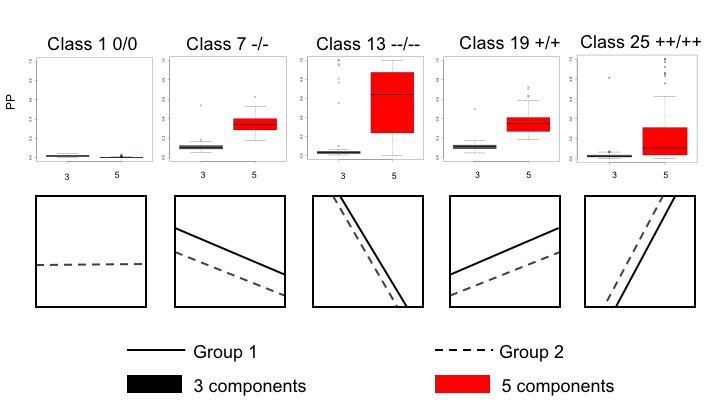


Supplementary Figure 10. Posterior probability distribution of no DC in 3- vs. 5- components. Since these are not cases of DC, the posterior probability of DC should be skewed to values close to 0.


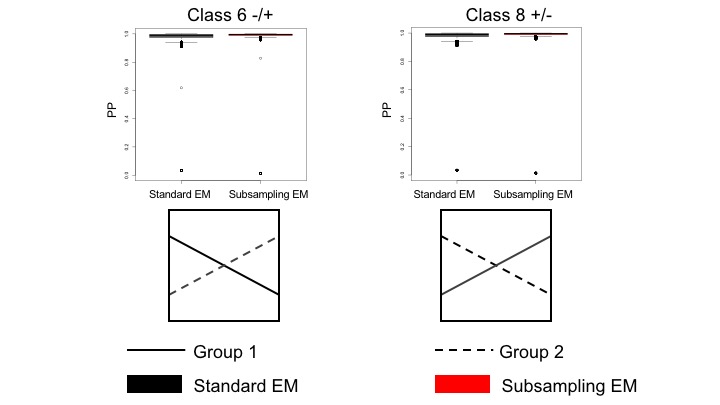


Supplementary Figure 11. Posterior probability distribution of cross DC in standard EM vs. subsampling EM. Since these are cases of DC, the posterior probability of DC should be skewed to values close to 1.


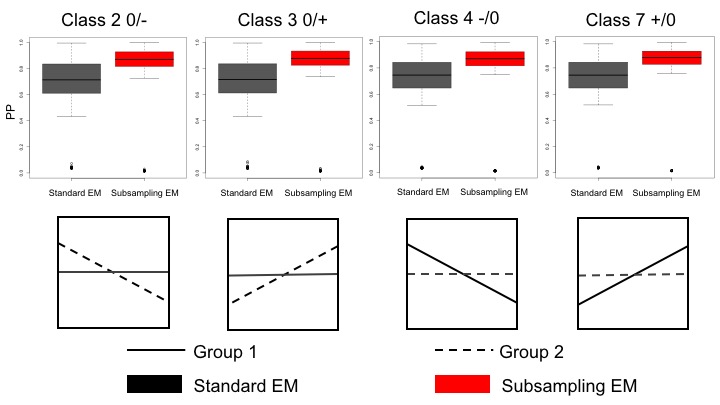


Supplementary Figure 12. Posterior probability distribution of disrupted DC in standard EM vs. subsampling EM. Since these are cases of DC, the posterior probability of DC should be skewed to values close to 1.


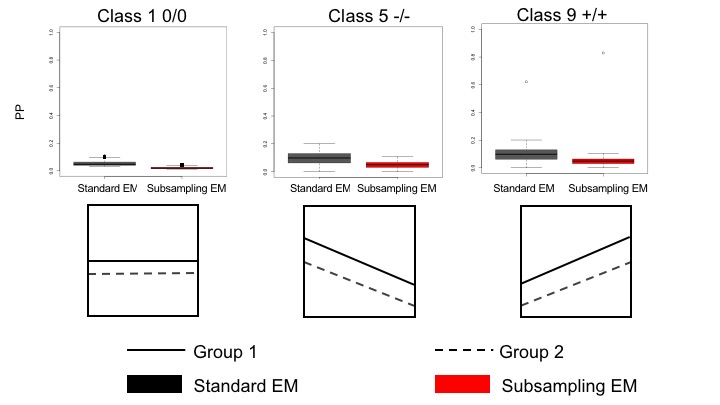


Supplementary Figure 13. Posterior probability distribution of no DC in standard EM vs. subsampling EM. Since these are not cases of DC, the posterior probability of DC should be skewed to values close to 0.

| treatment | statistic | hsa-  mir-107 | hsa-mir-150 | hsa-mir-152 | hsa-mir-191 | hsa-  mir-24-2 | hsa-  mir-374a | hsa-mir-574 | hsa-mir-454 |
| --- | --- | --- | --- | --- | --- | --- | --- | --- | --- |
| Correlation method comparison | | | | | | | | | |
| Spearman | rank | 4 | 1 | 72 | 135 | 85 | 7 | 368 | 40 |
|  | 1-PP | 0.0027 | 8.3e-4 | 0.0115 | 0.0149 | 0.0125 | 0.0039 | 0.0248 | 0.0087 |
|  | q-value | 0.0042 | 6.5e-4 | 0.0174 | 0.0224 | 0.0185 | 0.0056 | 0.0379 | 0.0125 |
| SparCC | rank | 2941 | 68 | 334 | 458 | 90 | 846 | 1868 | 631 |
|  | 1-PP | 0.032 | 0.007 | 0.0135 | 0.0153 | 0.0078 | 0.012 | 0.0266 | 0.0174 |
|  | q-value | 0.045 | 0.010 | 0.0191 | 0.0214 | 0.0190 | 0.017 | 0.0371 | 0.0241 |
| Pearson | rank | 428 | 41 | 427 | 717 | 3 | 919 | 102 | 1612 |
|  | 1-PP | 0.046 | 0.017 | 0.046 | 0.056 | 0.0056 | 0.062 | 0.025 | 0.078 |
|  | q-value | 0.065 | 0.024 | 0.065 | 0.079 | 0.0056 | 0.088 | 0.035 | 0.112 |
| BWMC | rank | 364 | 2 | 207 | 408 | 89 | 342 | 864 | 79 |
|  | 1-PP | 0.046 | 0.0066 | 0.037 | 0.049 | 0.026 | 0.045 | 0.066 | 0.025 |
|  | q-value | 0.065 | 0.0079 | 0.052 | 0.068 | 0.036 | 0.064 | 0.093 | 0.035 |
| DiffCorr | rank | 818 | 225 | 289 | 65 | 501 | 180 | 1225 | 718 |
|  | p-value | 6.96e-5 | 1.05e-5 | 1.55e-5 | 1.78e-6 | 3.45e-5 | 7.24e-6 | 1.18e-4 | 5.74e-5 |
|  | FDR | 0.289 | 0.158 | 0.182 | 0.085 | 0.233 | 0.136 | 0.328 | 0.271 |
| 3 component vs. 5 component | | | | | | | | | |
| 3 | rank | 4 | 1 | 72 | 135 | 85 | 7 | 368 | 40 |
|  | 1-PP | 0.0027 | 8.3e-4 | 0.0115 | 0.0149 | 0.0125 | 0.0039 | 0.0248 | 0.0087 |
|  | q-value | 0.0042 | 6.5e-4 | 0.0174 | 0.0224 | 0.0185 | 0.0056 | 0.0379 | 0.0125 |
| 5 | rank | 2067 | 363 | 51 | 32 | 276 | 1811 | 444 | 421 |
|  | 1-PP | 9.54e-4 | 1.45e-4 | 1.83e-5 | 1.40e-5 | 1.08e-4 | 8.12e-4 | 1.75e-4 | 1.67e-4 |
|  | q-value | 2.23e-3 | 3.16e-4 | 3.74e-5 | 3.15e-5 | 2.32e-4 | 1.89e-3 | 3.87e-4 | 3.68e-4 |
| Standard EM vs. Subsampling EM | | | | | | | | | |
| Standard | rank | 4 | 1 | 72 | 135 | 85 | 7 | 368 | 40 |
|  | 1-PP | 0.0027 | 8.3e-4 | 0.0115 | 0.0149 | 0.0125 | 0.0039 | 0.0248 | 0.0087 |
|  | q-value | 0.0042 | 6.5e-4 | 0.0174 | 0.0224 | 0.0185 | 0.0056 | 0.0379 | 0.0125 |
| Subsampling | rank | 4 | 1 | 81 | 151 | 50 | 12 | 398 | 37 |
|  | 1-PP | 6.4e-4 | 1.5e-4 | 0.0037 | 0.005 | 0.0028 | 0.0012 | 0.0087 | 0.0024 |
|  | q-value | 0.0010 | 0 | 0.0056 | 0.008 | 0.0043 | 0.0020 | 0.0138 | 0.0037 |

Supplementary Table 1. TCGA breast cancer biological validation. Ranks and 1-PP values are reported for the most significant result for the breast cancer miRNA paired with any gene.

| 1000 feature pairs | |
| --- | --- |
| 3-component mixture model | 3.9 seconds |
| 5-component mixture model | 10.6 seconds |
| 20,000 feature pairs | |
| Standard EM | 51.9 seconds |
| Subsampling | 27.9 seconds |

Supplementary Table 2. Run-time of Discordant in simulations on 3-component vs. 5-component mixture models and Standard EM vs. Subsampling.
